# Editorial: New theories, models, and AI methods of brain dynamics, brain decoding and neuromodulation

**DOI:** 10.3389/fnins.2023.1302505

**Published:** 2023-12-12

**Authors:** Yuzhu Guo, Yang Li, Hua-Liang Wei, Yifan Zhao

**Affiliations:** ^1^School of Automation Science and Electrical Engineering, Beihang University, Beijing, China; ^2^BAIoT Brain-Computer Intelligence Joint Laboratory, Beijing, China; ^3^Department of Automatic Control and System Engineering, University of Sheffield, Sheffield, United Kingdom; ^4^Centre for Life-Cycle Engineering and Management, Cranfield University, Cranfield, United Kingdom

**Keywords:** brain dynamics, brain connectivity, neuromodulation, brain decoding, neural coupling

The human brain is highly dynamic and complex, supporting a remarkable range of functions by dynamically integrating and coordinating different brain regions and networks across multiple spatial and temporal scales. Research on the human brain has become truly interdisciplinary involving medicine, neurobiology, engineering, and related fields. A thorough understanding of the mechanisms of neuromodulation actions is urgently needed for stimulation parameters optimization, response prediction, and consistent therapy. This Research Topic aims to combine top-down and bottom-up methods to produce robust results that allow for a meaningful interpretation in terms of the underlying brain dynamics with an emphasis on brain decoding and neuromodulation.

Since the nonlinear, non-stationary, and complex couplings in brain activity, extremely rich information, including temporal, spatial, frequency, phase, and connectivity features, is embedded in every single measurement (Cao et al., 2022). Many methods are dedicated to extracting specific features from the measurement. Even though more and more end-to-end deep models have been utilized for brain activity decoding, including convolutional neural networks, graphical neural networks, attention models, capsule networks, generative models, and so on, revealing the underlying mechanisms is essential for clinical practices (Li et al., 2023), especially, neuromodulation. Hence, an important alternative is to study these features as a whole and study the complex couplings among a wide range of brain activity (Li et al., 2022).

The collection of articles in this Research Topic showcases the diversity of theoretical and empirical developments across a wide spectrum of brain dynamics research into complex couplings. Although this Research Topic only accepts four articles following the review process, it still covers a surprisingly wide range of approaches. Liu et al. studied the cross-domain data augmentation and showed that combining spatial-temporal features can improve the richness of generated data and contribute to the identification of brain disorders; Kim et al. focused on the cross-frequency couplings (CFC) and the CFC- transcranial alternating current stimulation (CFC-tACS) was used to improve working memory performance and resulted in a significantly reduced response time; de Freitas Zanona et al. studied inter-stimulus coupling and showed that the somatosensory cortex (S1) repetitive transcranial magnetic stimulation (rTMS) and sensory stimulation (SS) alone or in combination the S1 excitability was changed, but only their combination increased primary motor cortex (M1) excitability; Guo et al. revealed the connections between retinal microvascular changes and NMOSD.

In summary, this Research Topic highlights multiple methods for capturing brain dynamics and coupling analysis with high potential in a wide range of applications, such as brain disorder identification (Liu et al.), improvement of working memory (Kim et al.), treatment of stroke (de Freitas Zanona et al.) and biomarker discovery (Guo et al.), and so on. The brain dynamics and coupling analyses, especially, have far-reaching implications on neuromodulation.

## Author contributions

YG: Writing—original draft. YL: Writing—review & editing. H-LW: Writing—review & editing. YZ: Writing—review & editing.
